# Why all MODY variants in transcription factor genes are dominantly inherited

**DOI:** 10.3389/fgene.2025.1690468

**Published:** 2025-11-20

**Authors:** Roman Zug

**Affiliations:** 1 Institute of Organismic and Molecular Evolution (iomE), Johannes Gutenberg University Mainz, Mainz, Germany; 2 Institute for Quantitative and Computational Biosciences (IQCB), Johannes Gutenberg University Mainz, Mainz, Germany

**Keywords:** monogenic diabetes, cell fate, haploinsufficiency, dosage sensitivity, positive feedback, cooperativity, bistability, incomplete penetrance

## Abstract

Maturity-onset diabetes of the young (MODY) is an autosomal dominant form of monogenic diabetes, frequently caused by heterozygous loss-of-function variants in transcription factor (TF) genes. Why are MODY variants in TF genes dominantly inherited? Here I present a systems biology-based explanation. The fact that MODY-associated TFs are master regulators of pancreatic β cell fate suggests that pathogenic variants cause defects in cell fate determination. From a systems biology perspective, cell fate defects are based on disrupted bistability, a crucial feature of dynamical systems to make binary choices. Bistability requires both positive feedback and ultrasensitivity, the latter often in the form of cooperativity. MODY-associated TFs exhibit both features, which not only allows for bistability, but also makes these TFs extremely dosage sensitive, which explains why heterozygous loss of function is sufficient to cause a disease phenotype. A review of the literature strongly supports this hypothesis. Moreover, the hypothesis also helps to explain why incomplete penetrance is such a pervasive feature of MODY-associated variants in TF genes.

## Introduction

Maturity-onset diabetes of the young (MODY) is a rare inherited form of diabetes caused by mutations in a single gene and characterized by an early onset, typically before the age of 25 years. MODY represents the most common form of monogenic diabetes and is due to impaired development and function of pancreatic β cells, resulting in deficient secretion of insulin. MODY is inherited predominantly in an autosomal dominant mode, which is remarkable because other forms of monogenic diabetes do not show this inheritance pattern (Bonnefond et al., 2023). Why are most MODY variants dominantly inherited? In an attempt to address this issue, Li et al. (2022) asked: “Could it be because of some biological property of the insulin-secreting pancreatic β cells that makes them susceptible to the deleterious effects of heterozygous but not homozygous variants?” Interestingly, however, rather than giving an answer to this question, Li et al. proposed that autosomal recessive forms of MODY “are at least as common as the dominant ones, but have not been discovered yet”. In their view, the preponderance of autosomal dominant MODY does not have a biological reason, but rather reflects our inability to detect recessive variants. While this is theoretically possible, I here argue that there is indeed a plausible biological reason why MODY is mostly dominant. In the following sections I will outline the hypothesis step by step, together with empirical evidence. Finally, I discuss why the hypothesis also helps to explain incomplete penetrance of MODY variants, and I disprove the idea that homozygous variants are less deleterious.

## Most MODY cases are caused by variants in master transcription factor (TF) genes

**FIGURE 1:**
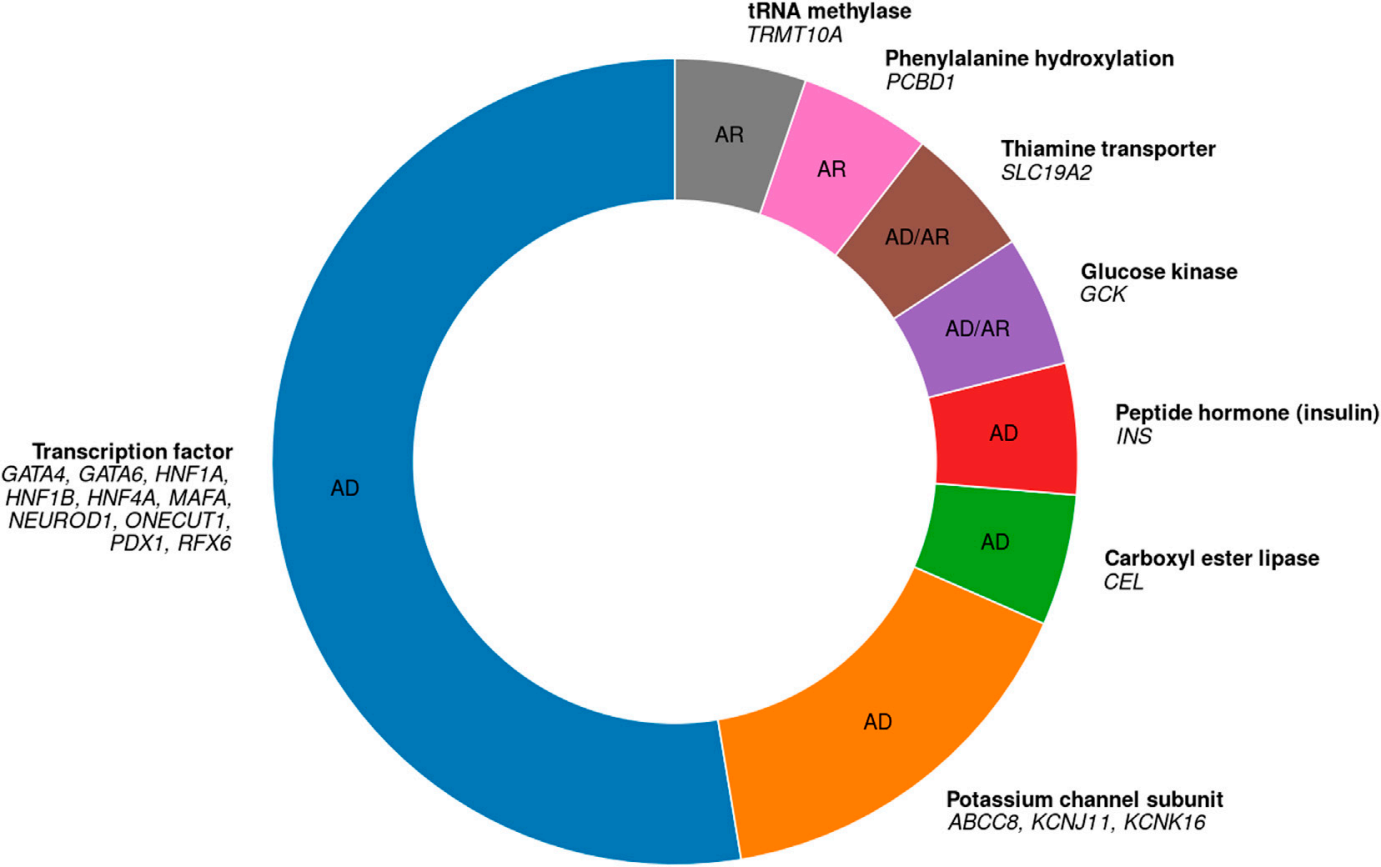
All 19 known autosomal MODY genes, their protein functions, and their inheritance modes. 10 genes code for transcription factors, 3 for potassium channel subunits, and one gene, respectively, for each of the other categories. AD, autosomal dominant; AR, autosomal recessive. Based on Bonnefond et al. (2023) and Sriram et al. (2025).

Although the protein products of MODY genes serve a variety of molecular functions, by far the largest functional group comprises transcription factors (TFs) (Figure 1). These TFs act as master regulators of pancreatic development, and of β cell differentiation and function in particular (Arda et al., 2013; Conrad et al., 2014; Dassaye et al., 2016; Wortham and Sander, 2021). Therefore, MODY cases that are due to impaired pancreas and β cell development, caused by variants in master TF genes, should be considered developmental disorders (Zug, 2022).

Out of 19 autosomal genes in which MODY-causing variants are known, 10 code for TFs (Figure 1) (Bonnefond et al., 2023). These TF genes are given in Table 1. Note that I do not include two other TF genes, *KLF11* and *PAX4* (nor the genes *APPL1*, *BLK*, and *WFS1*), because there is insufficient evidence that variants in these genes actually cause MODY (Laver et al., 2022; Sriram et al., 2025). Of the 10 known MODY-associated TF genes, the three most common ones alone are estimated to account for more than two-thirds of all MODY cases: *HNF1A* (52%), *HNF4A* (10%), and *HNF1B* (6%) (Shields et al., 2010).

**TABLE 1:** Features of transcription factor (TF) genes in which MODY-causing variants have been identified. All variants exhibit autosomal dominant inheritance.

TF gene	Haploinsufficiency (intolerance to heterozygous LOF) as the cause of MODY	Pancreatic development, β cell fate and function	Positive feedback	Cooperativity	Estimated strength of selection against heterozygous LOF^a^
*GATA4*	Shaw-Smith et al. (2014)	Carrasco et al. (2012); Xuan et al. (2012)	Arda et al. (2013)	Charron et al. (1999); Xin et al. (2006)	0.115 (extreme selection)
*GATA6*	Bonnefond et al. (2012)	Carrasco et al. (2012); Xuan et al. (2012)	Meng et al. (2018)	Charron et al. (1999); Xin et al. (2006); Chia et al. (2019)	0.318 (extreme selection)
*HNF1A*	Yamagata et al. (1996b)	Qian et al. (2023); Ng et al. (2024)	Ferrer (2002); Hansen et al. (2002)	Cujba et al. (2022)	0.084 (strong selection)
*HNF1B*	Horikawa et al. (1997)	Cujba et al. (2022)	Arda et al. (2013); De Vas et al. (2015)	Cujba et al. (2022)	0.251 (extreme selection)
*HNF4A*	Yamagata et al. (1996a)	Ng et al. (2019), Ng et al. (2024)	Ferrer (2002); Hansen et al. (2002)	Jiang and Sladek (1997); Lu et al. (2008)	0.051 (strong selection)
*MAFA*	Iacovazzo et al. (2018)	Olbrot et al. (2002); Nishimura et al. (2015)	Raum et al. (2006)	Zhao et al. (2005)	0.008 (strong selection)
*NEUROD1*	Malecki et al. (1999)	Gu et al. (2010); Jia et al. (2015); Bohuslavova et al. (2023)	Arda et al. (2013)	Zhao et al. (2005); Jia et al. (2015)	0.100 (extreme selection)
*ONECUT1*	Philippi et al. (2021)	Heller et al. (2021)	Arda et al., 2013; De Vas et al., 2015	Henley et al. (2016)	0.081 (strong selection)
*PDX1*	Stoffers et al. (1997a)	Gao et al. (2014)	Raum et al. (2006); Shih et al. (2015)	Zhao et al. (2005); Shih et al. (2015); Henley et al. (2016); Bastidas-Ponce et al. (2017)	0.005 (strong selection)
*RFX6*	Patel et al. (2017)	Piccand et al. (2014); Ibrahim et al. (2024)	He et al. (2022)	Churchill et al. (2017)	0.037 (strong selection)

^a^ Estimated *s*_het_ values taken from Zeng et al. (2024). Under the strong selection regime (10^−3^ > *s*_het_ < 10^−1^), heterozygous LOF has a fitness effect on par with the strongest selection measured for common variants. Under extreme selection (*s*_het_ > 10^–1^), fitness effects are equivalent to a >10% chance of embryonic lethality (Zeng et al., 2024).

## Haploinsufficiency of master TF genes as a cause of developmental disorders

All known MODY-causing variants in TF genes show dominant inheritance. Why is that? In order to answer this question, let us look at these variants in more detail. All MODY-associated variants in TF genes cause loss-of-function (LOF). This is not surprising, as most mutations cause LOF. What is surprising, though, is that LOF of a single allele is sufficient to cause a clinical phenotype. In other words, a 50% reduction in gene expression is not tolerated. This pronounced dosage sensitivity is called haploinsufficiency. Strikingly, all MODY-associated TF genes are haploinsufficient and hence intolerant to heterozygous LOF variants (Table 1). Haploinsufficiency is a manifestation of genetic dominance, as a phenotype is already visible in the heterozygous state (Zschocke et al., 2023). Haploinsufficiency represents a particularly strict form of gene essentiality, which can be defined as a considerable reduction in organismal fitness associated with a gene’s LOF (Bartha et al., 2018). Accordingly, in haploinsufficient genes, there is strong negative selection even against heterozygous LOF variants, reducing the frequency of such variants in the population (‘selective constraint’) (Zeng et al., 2024). Haploinsufficiency is a hallmark of master regulator TF genes and can lead to a plethora of developmental disorders, MODY being one of them (Seidman and Seidman, 2002; Zug, 2022). Most often, MODY is caused by LOF variants in the coding regions of TF genes, but it can also be due to LOF variants in the cis-regulatory elements (CREs) of these genes, as has been shown, for example, for *HNF1A* (Gragnoli et al., 1997) and *HNF4A* (Hansen et al., 2002).

## Bistability and its disruption through TF haploinsufficiency

**FIGURE 2:**
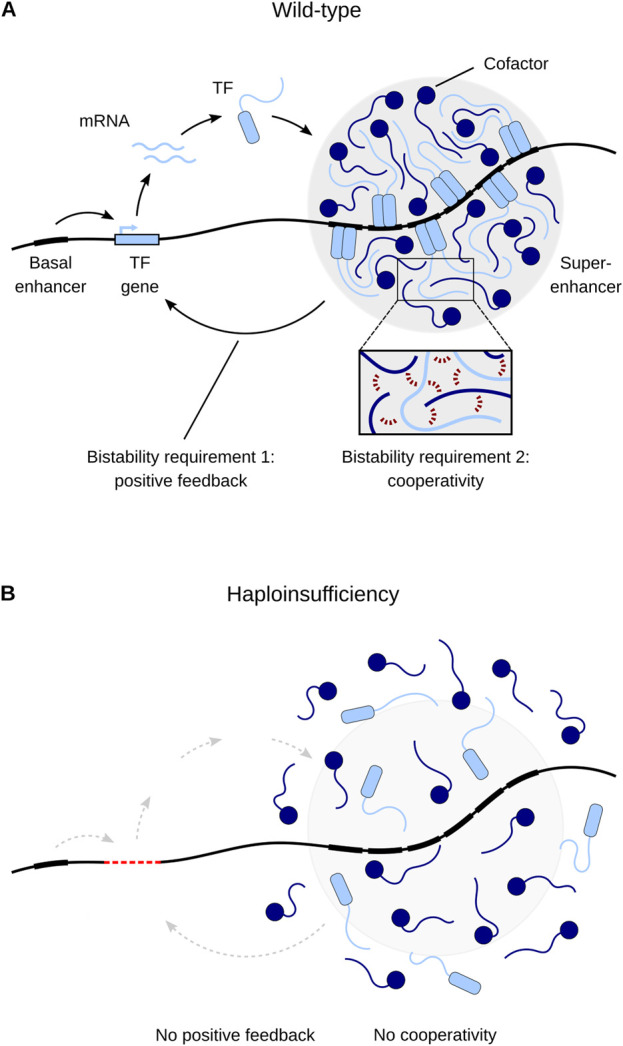
A model of transcriptional regulation of cell fate and its misregulation due to haploinsufficiency. **(A)** Master transcription factors (TFs) maintain their own expression through positive feedback by cooperatively binding to their own super-enhancers. Cooperative binding occurs through multivalent interactions between TFs and cofactors (inset, red dashed lines). **(B)** Haploinsufficiency (here caused by TF gene deletion) disrupts positive feedback and cooperativity and thus leads to disease. Adapted from Zug (2022).

Why are heterozygous LOF variants in TF genes (or their CREs) sufficient to cause MODY? In other words, why are these TFs so dosage-sensitive? Building upon earlier work (Wilkie, 1994; Veitia, 2002; Johnson et al., 2019), I have recently proposed the hypothesis that developmental disorders caused by TF haploinsufficiency result from defects in cell fate determination, which can be traced to disrupted bistability in the underlying gene regulatory network (Zug, 2022). Bistability is a crucial feature of dynamical systems that are able to make binary choices such as cell fate decisions. Bistability means that a system can be resting in two alternative stable states but not in intermediate states, resulting in switch-like threshold effects. The threshold corresponds to an unstable steady state separating the two stable steady states. A system is able to generate bistability if its components engage both in positive feedback and ultrasensitivity (Ferrell, 2002). Positive feedback prevents the system from resting in intermediate states. Ultrasensitivity means that an increase in the input signal first has little effect, but then produces higher and higher levels of output, as represented by a steep sigmoidal curve. Ultrasensitivity filters small stimuli out of the feedback loop, allowing the system to have a stable off-state (Ferrell, 2002), and often comes in the form of cooperativity (Zhang et al., 2013). Bistable switches based on positive feedback and cooperativity are a pervasive regulatory motif underling cell fate determination. Cell fate is mainly controlled by the assembly of master regulators at cell-type-specific enhancers, often called super-enhancers (Figure 2A). Genes associated with super-enhancers include those encoding the master regulators themselves, thus establishing autoregulatory positive feedback loops. Super-enhancers assemble a high density of master regulators, allowing for extensive cooperative TF-DNA binding (e.g., via dimerization). The requirement of both positive feedback and cooperativity for proper cell fate determination helps to explain the distinct dosage sensitivity (that is, haploinsufficiency) of master regulators: it is the high level of cooperativity (an instantiation of ultrasensitivity) that makes the positive feedback loops particularly sensitive to changes in TF concentration. Therefore, heterozygous LOF variants that disrupt positive feedback or cooperativity are sufficient to interfere with proper cell fate determination and eventually lead to developmental disorders such as MODY (Figure 2B) (Zug, 2022). Although bistability has been invoked before to explain MODY etiology (in the context of the HNF1A–HNF4A positive feedback loop: Ferrer, 2002; Kaci et al., 2024), these studies ignore cooperativity, failing to account for both requirements of bistability.

## Supporting evidence with respect to MODY

Here I collect evidence in support of the hypothesis that heterozygous LOF variants in MODY-associated TF genes disrupt positive feedback or cooperativity and thus cause the disease. As shown in Table 1, all known MODY-associated TF genes (1) are haploinsufficient, (2) are master regulators of pancreatic development, and of β cell fate and function in particular, (3) engage in positive feedback and cooperativity, and (4) exhibit strong or extreme selection against heterozygous LOF, as estimated by a powerful Empirical Bayes approach (Zeng et al., 2024). Moreover, for *HNF1A* and *HNF4A*, disruption of positive feedback (Hansen et al., 2002) and of cooperativity (Hua et al., 2000; Singh et al., 2019) has been identified as the cause of MODY. Taken together, this evidence strongly supports the hypothesis that heterozygous LOF variants in master regulators of β cell fate are sufficient to disrupt TF positive feedback or cooperativity and thus cause MODY. This explains why MODY-associated variants in TF genes are dominantly inherited.

## A better understanding of incomplete penetrance of MODY-associated TF genes

**FIGURE 3:**
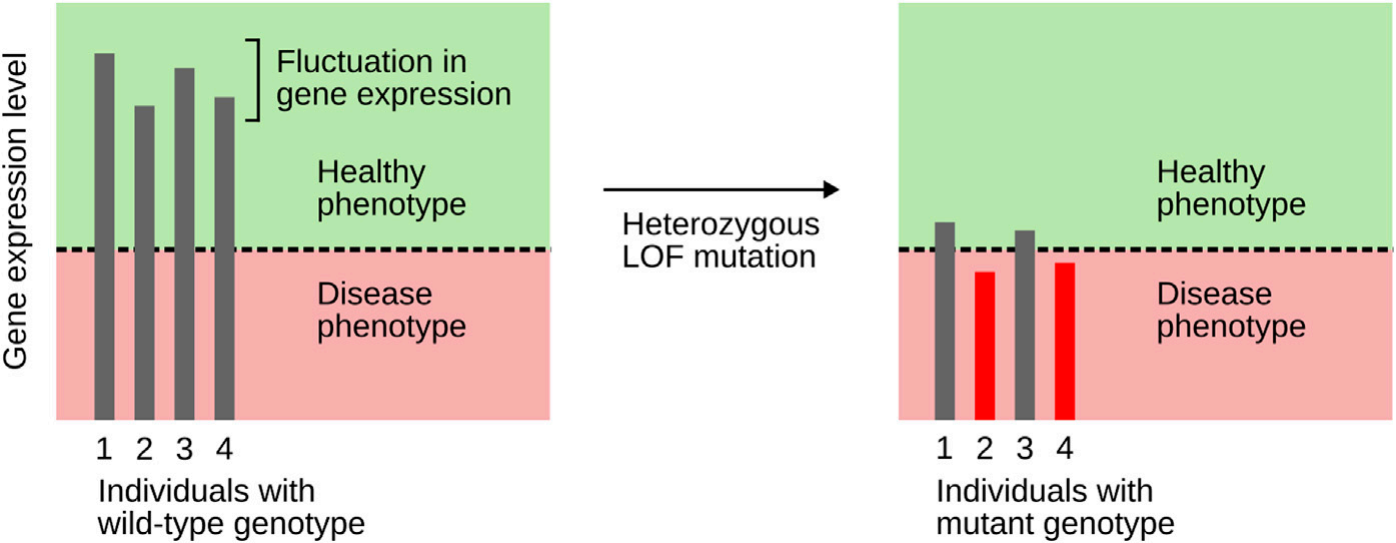
An explanation of incomplete penetrance, based on the combined action of stochastic fluctuation in gene expression and a threshold effect. The latter results from TF genes engaging in positive feedback and cooperativity. Heterozygous LOF mutations bring expression levels close to the threshold. Only individuals whose gene expression lies below the threshold (here, individuals 2 and 4) will show a disease phenotype. Based on Hobert (2010).

The hypothesis outlined above also helps to better understand why not every individual carrying a pathogenic variant actually develops the disease, a phenomenon termed incomplete penetrance (Kingdom and Wright, 2022). Many MODY-associated TF genes show incomplete penetrance, e.g., *HNF1A*, *HNF1B*, *HNF4A*, *NEUROD1*, *PDX1*, and *RFX6* (Mirshahi et al., 2022; Li et al., 2023; Sharp et al., 2025; Sriram et al., 2025). Even though incomplete penetrance can be caused by a range of factors, it has a strong genetic basis (Kingdom and Wright, 2022). Goldschmidt (1938) explained incomplete penetrance by assuming stochastic fluctuation in gene expression combined with some threshold effect. The stochastic nature of gene expression is now well established (Raj and van Oudenaarden, 2008). Goldschmidt’s postulated threshold effect can be readily explained as well, at least with respect to master regulator TFs: as outlined above, these TFs engage in positive feedback and cooperativity, which allows for bistability and, thus, threshold effects. Heterozygous LOF variants bring gene expression levels close to the threshold, but only those variants for which gene expression happens to lie below the threshold will elicit a disease phenotype (Figure 3). This idea explains incomplete penetrance of variants in TF genes, including those associated with MODY (Zug, 2022).

## Homozygous LOF variants generally show worse outcomes than heterozygous variants

Lastly, I would like to address the assumption made by Li et al. (2022) that β cells are susceptible to heterozygous but not homozygous variants. I argue that this assumption is wrong, at least with respect to TF genes. The reason is that, in TF genes, variants in the homozygous state have generally more severe consequences than in the heterozygous state, a phenomenon termed semi-dominance (Zschocke et al., 2023). In many MODY-associated TF genes, homozygous LOF variants are generally thought to be embryonically lethal or result in early mortality, e.g., in *GATA4* (Kuo et al., 1997), *GATA6* (Morrisey et al., 1998), *HNF1A* (Harries et al., 2009), *HNF1B* (Barbacci et al., 1999), *HNF4A* (Chen et al., 1994), and *ONECUT1* (Philippi et al., 2021). In other MODY-associated TF genes, homozygous variants exist but cause severe syndromic and usually neonatal diabetes, such as in *MAFA* (Iacovazzo et al., 2018), *NEUROD1* (Rubio-Cabezas et al., 2010), *PDX1* (Stoffers et al., 1997b) and *RFX6* (Smith et al., 2010). Therefore, the question is not why MODY-associated TF genes are not susceptible to homozygous variants (they are very much so), but rather why homozygous variants are tolerated at all (at least in some genes), given the generally high intolerance of these genes even to heterozygous variants.

## Conclusion

LOF variants in TFs controlling pancreatic β cell fate are a common cause of MODY. To understand the dominant inheritance of such variants, I have adopted a systems biology perspective. I have shown that MODY-associated TFs are involved in positive feedback and cooperativity, which makes them extremely dosage-sensitive and thus explains why even heterozygous LOF is not tolerated, resulting in dominance. The proposed hypothesis also helps to explain incomplete penetrance, which is widespread in MODY, and thus advances our understanding of the most common form of monogenic diabetes. Future studies should gather further empirical evidence showing that MODY is caused by disrupted TF cooperativity or positive feedback (beyond *HNF1A* and *HNF4A*). Another important issue for future work is to investigate what distinguishes those TF genes that are able to tolerate homozygous LOF variants. It will also be interesting to elucidate how functioning of the bistable switch motif is affected by polygenic background, which can substantially modify MODY penetrance and expressivity (Murray Leech et al., 2025).
